# Physical workload and glycemia changes during football matches in adolescents with type 1 diabetes can be comparable

**DOI:** 10.1007/s00592-019-01371-0

**Published:** 2019-06-04

**Authors:** Andrzej Gawrecki, Arkadiusz Michalak, Szymon Gałczyński, Iwona Dachowska, Dorota Zozulińska-Ziółkiewicz, Agnieszka Szadkowska

**Affiliations:** 1 Poznan University of Medical Sciences, Poznan, Poland; 2 Department of Pediatrics, Diabetology, Endocrinology and Nephrology, Medical University of Lodz, ul. Sporna 36/50, 91-738 Lodz, Poland; 3 The Academic Laboratory of Movement and Human Physical Performance DynamoLab, Medical University of Lodz, Lodz, Poland; 4 Department of Pediatrics, Oncology and Hematology, Medical University of Lodz, Lodz, Poland

**Keywords:** Type 1 diabetes, Exercise, Football, GPS tracking, HR monitoring

## Abstract

**Aims:**

To analyze physical performance and diabetes-related outcomes in adolescents with type 1 diabetes (T1DM) during two semi-competitive football matches utilising precise physical activity monitoring.

**Methods:**

The study was conducted during an annual summer camp for adolescents with T1DM. After physical examination and glycated hemoglobin measurement, 16 adolescent players completed Cooper’s 12-min running test and, in the following days, took part in two football matches while wearing heart rate (HR) monitors coupled with global positioning system (GPS) tracking.

**Results:**

Both matches were comparable in terms of covered distances, number of sprints, achieved velocities and heart rate responses. During both games, capillary blood lactate increased significantly (Match 1: 1.75 ± 0.16–6.13 ± 1.73 mmol/l; Match 2: 1.77 ± 0.18–3.91 ± 0.63 mmol/l, *p* = 0.004). No significant differences in blood glucose were observed between the matches (*p* = 0.83) or over each match (*p* = 0.78). Clinically significant hypoglycemia (< 54 mg/dl) occurred in two children during the first match. None of the players experienced severe hypoglycemia. Despite similar workloads, players consumed significantly less carbohydrates during Match 2 [median difference: − 20 g (25–75%: − 40 to 0), *p* = 0.006].

**Conclusions:**

HR monitoring and GPS-based tracking can effectively parameterize physical activity during a football match. In T1DM patients, exercise workload and glycemic changes during similar matches are comparable, which provides an opportunity to develop individual recommendations for players with T1DM.

**Electronic supplementary material:**

The online version of this article (10.1007/s00592-019-01371-0) contains supplementary material, which is available to authorized users.

## Introduction

Physical activity offers great benefits for patients with type 1 diabetes (T1DM), and thanks to advances in pharmacotherapy and implementation of new technological solutions and education, different sports disciplines are now becoming more accessible for them. T1DM patients currently participate in all types of sports activities, even extreme sports [1–3]. However, T1DM patients who engage in long or intensive exercise still face a challenge of high glycemic variability and, more importantly, an increased risk of hypo- and sometimes hyperglycemia, which can limit an athlete’s motivation and performance. Therefore, it is crucial to prevent acute and potentially serious complications of these conditions (severe hypoglycemia and ketoacidosis) to enable T1DM patients to safely participate in sport trainings and competitions.

Sporting activities are particularly important for children and adolescents with T1DM, for whom physical activity is an integral element of somatic development [4]. However, maintaining normoglycemia during exercise in this age-group is very challenging due to high frequency of impromptu activities and changeable workloads. Although continuous subcutaneous insulin infusion (CSII) with modern personal insulin pumps has improved precision of adjusting insulin doses to patients’ physical effort and the use of continuous glucose monitoring systems has provided detailed insight into glucose dynamics, maintaining safety during exercise still requires caregivers’ and patients’ commitment and thoughtful physicians’ advices.

Several guidelines for T1DM patients regarding exercise have been recently published [5–8]. Although the basic aspects of activity management (i.e., insulin dose reduction and additional carbohydrates consumption) are well founded, many recommendations lack extensive scientific evidence and are based on singular studies or experts’ opinions [7]. It has been proposed that besides the above-mentioned conventional factors, multitude of additional circumstances may affect glycemic response to exercise, including the amount of circulating insulin (“insulin on board”), the glucose concentration trends, composition and volume of a meal consumed before exercise, and also the intensity, type and duration of exercise [8]. Pathophysiology of T1DM can partly explain why the generally accepted rules do not always allow patients to achieve and maintain normal blood glucose levels. In most patients, insulin concentration in blood can be too high with respect to glucose utilization, because insulin level cannot be suddenly reduced during physical exertion as it is in healthy people [9]. Sometimes situation is opposite: The amount of circulating blood insulin, especially during maximum, anaerobic efforts, may be too low which may result in prominent hyperglycemia. Moreover, studies have shown that each patient has specific physical activity-related needs, and this is why recommendations should be individually tailored [10]. However, to date, these adjustments were made mostly based on patient or practitioner experience [12].

To power up the creation of more effective guidelines, we propose to improve the quality of collected data on physical activity by precise quantification of exercise workloads. For in the field sports practicing, this might be achieved by using heart rate (HR) monitoring and global positioning system (GPS) tracking, which provide objective information on workload intensity and kinematics (covered distance, maximum and mean velocity). Coupled with blood glucose measurements or data from continuous glucose monitoring (CGM), these systems could help in designing repeatable training plans which enable that predictable glycemic excursions can be managed accordingly. In children, such approach may also uncover previously unrecognized patterns of exercise–glycemia relations.

GPS tracking and HR monitoring are already widely used in professional football (US soccer) [11], which is the sport that teenagers in many countries practice most commonly. The intensity of physical effort during a football match is hard to predict, and retrospective analysis after such a match is also difficult to perform, which makes blood glucose control during football trainings and competitions a considerable challenge. The aim of the study was to analyze physical performance and diabetes-related outcomes in adolescents with T1DM during semi-competitive football matches. Therefore, we performed a proof-of-concept study assessing feasibility of using GPS-tracking and HR-monitoring technology during two football matches played by teenagers with T1DM and we attempted to characterize the intensity of physical activity and glycemic excursions during the games.

## Materials and methods

The study was conducted during an annual summer camp for children and adolescents with T1DM. Males aged 12–17 years were recruited to play two football matches under HR monitoring and GPS tracking. Children remained under medical supervision for the duration of the camp (July 11 to July 24, 2017). The study protocol was approved by the Ethics Committee of the Medical University of Lodz (NO RNN/195/17/KE) and therefore was performed in accordance with the ethical standards laid down in the 1964 Declaration of Helsinki and its later amendments. Informed written consent was obtained from parents or participants > 16 years old, and informed assent from the rest of the children.

All participants were treated with functional intensive insulin therapy by CSII or multiple daily injections (MDIs). During the camp and while playing the matches, all children performed self-measurements of capillary blood glucose to adjust insulin doses. They also used continuous glucose monitoring (Guardian Connect CGM, Medtronic, Northridge, CA), sensor-augmented insulin pumps (MiniMed Real-Time, MiniMed VEO, MiniMed 640G, Medtronic, Northridge, CA) or flash glucose monitoring (Libre, Abbott Diabetes Care, Alameda, CA) to continuously record glucose levels in the interstitial tissue and to have access to glucose concentration trend arrows when necessary.

Physicians obtained medical history and performed a physical examination for all participants. Body height and weight were measured to calculate body mass index (BMI). Capillary blood samples were collected for HbA1c assessment [D-10 Hemoglobin A1c Program (Bio-Rad Laboratories, Hercules, CA, Bio-Rad, Marnes-la-Coquette, France)].

At the beginning of the camp, all participants completed Cooper’s 12-min running test with chest-strapped heart rate (HR) monitors according to the protocol previously described to assess maximum heart rate and physical capacity [13, 14].

Two semi-competitive football matches (each lasting 80-min + a 10-min break) were organized on the 70 × 90 m football field. The matches were played within 4 days, at the same time of a day. Twenty-two participants agreed to take part in the study. Two teams of 11 children (nine playing and two in reserve) were appointed. Out of 18 players from the initial team, 16 (excluding goalkeepers) wore HR monitors coupled with GPS tracking, which allowed for continuous tracking of each player’s position and movement (Polar Team Pro System, Polar Electro OY, Kempele, Finland). Data collected for each participant included distance covered, mean and maximum velocity achieved, number of sprints performed and heart rate response. Sprints were defined as accelerations of ≥ 2 m/s^2^. Retrospective analysis of physical activity workload was performed with the Polar Flow, Polar Electro OY (Kempele, Finland) software.

### Diabetes control during matches

Preparation for the matches and the matches themselves were supervised by six medical staff members. Before the breakfast at 8.00 in the morning, children administered insulin according to carbohydrates exchange factor and correction factor without any dose reduction. The matches began 2.5 h later.

Before the matches, during the breaks, and at the end of each match, blood glucose (BG) was measured using a Contour Plus One Glucose meter (Ascensia Diabetes Care, Basel, Switzerland). The BG target range set in the study protocol was 100–250 mg/dl. When BG was < 100 mg/dl, participants received 10–25 g of simple carbohydrates depending on CGM glucose trend arrows and body weight. When BG was from 100 to 150 mg/dl, oral carbohydrates were not administered unless CGM glucose trend arrows showed glucose decrease. If the BG before or at the interval of the match was > 250 mg/dl, the correction dose was administered. (Regular correction dose calculated based on sensitivity factor was reduced by 50%.) If BG was > 300 mg/dl, blood beta-hydroxy-butyrate concentration was measured with Optium Xido™ test strips (Abbott Diabetes Care, Alameda, CA). In children treated with CSII, insulin pumps were disconnected from their bodies for the time of the matches. In children treated with MDI, basal insulin dose administered in the evening before matches was not reduced.

Capillary lactate concentrations were assessed before and after each match (Lactate Scout, EKF Diagnostics, Germany) using the enzymatic-amperometric method (SensLab GmbH, Leipzig Germany). All data regarding monitored and measured parameters, insulin doses, and the amount of carbohydrates consumed were recorded during the games by the medical team in participants’ monitoring charts.

### Statistics

Player characteristics are presented as mean ± standard deviation. HRs achieved during football matches were recalculated as % of maximum HR presented during Cooper’s test to avoid age-specific differences in raw HR. The authors acknowledge that the studied sample of adolescents was relatively small, which could result in underpowered analyses; however, some precautions were taken to provide valid results. The changes in BG and lactate concentration were assessed using ANOVA with repeated measures and paired design, which accounted for the fact that the same players participated in both games. Comparisons between the games were made with paired *t* test or Wilcoxon’s paired test, and the effects were presented as mean (± SD) or median (+ interquartile range) difference for each player. This allowed for the assessment of repeatability of exercise workload during two matches for the same player.

## Results

Out of 16 monitored players, two suffered minor injuries, and as a result, 14 adolescents took part in both matches and provided complete data eligible for analysis. Their mean age was 14.9 ± 1.4 years and mean diabetes duration was 7.2 ± 3.9 years. Eleven (79%) of them were treated with CSII and the remaining three used MDI. Their mean HbA1c concentration was 7.2 ± 0.6% (52 ± 0.32 mmol/mol), indicating good glycemic control. The players’ mean BMI *z*-score was 0.57 ± 0.87.

**Fig. 1:**
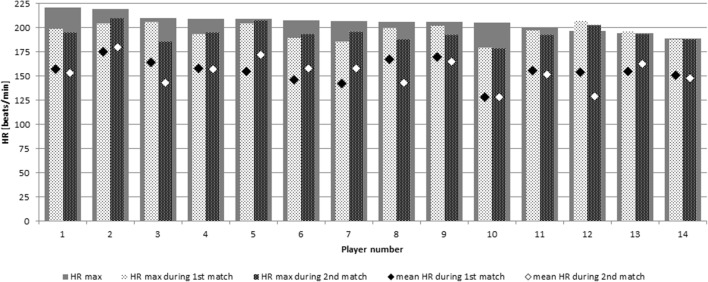
Maximal heart rates (HRs) measured for each participant during Cooper’s test and the figure contrasts them with their mean and maximal HR achieved during the first and second matches. Please note that most players reached HRs within 5 beats/min of their individual maximum during play. Player nos. 12 and 13 surpassed their maximal HRs, which indicated their underperformance during Cooper’s test

During Cooper’s test, participants presented average physical capacity as measured by distances (in meters), and they covered over 12 min expressed in sex- and age-adjusted *z*-scores (mean *z*-score − 0.04 ± 0.57, which corresponded to 49th ± 20 percentile for the reference population). Physical capacity did not correlate with glycemic control expressed as HbA1c (*r* = − 0.13, *p* = 0.66). A significant correlation was found between physical capacity and age (*r* = − 0.53, *p* = 0.05) and between physical capacity and BMI *z*-score (*r* = − 0.7, *p* = 0.006). Maximum HR reached by players during Cooper’s test ranged from 189 beats/min to 221 beats/min; two players surpassed their Cooper’s test maximum HR during either of matches. Mean HR achieved during matches was from 62 to 84% of players’ individual maximum Cooper’s test HR, and maximum HR registered during matches were comparable to individual Cooper’s test maximum HR values (Fig. 1).

Both matches provided comparable workloads for each player in terms of distance covered, number of sprints performed, mean and maximum velocity achieved and HR response (Table 1, Supplementary Fig. 1). The only relationship between the measured parameters of workload during matches, age, BMI *z*-score, glycemic control measured by HbA1c and physical capacity estimated by Cooper’s test was a positive correlation between age and maximum velocity achieved during the first match (*r* = 0.62, *p* = 0.017).

**Table 1:** Players’ performance during both matches

	First match (mean ± SD)	Second match (mean ± SD)	Difference by player (mean ± SD)	*p* value
Covered distance (km)	6.3 ± 1	6.3 ± 1.5	0.1 ± 1.2	0.58
No. of sprints	44 ± 17	42 ± 19	2 ± 14	0.58
Mean velocity (km/h)	3.9 ± 0.6	4.2 ± 0.9	− 0.2 ± 0.7	0.26
Max velocity (km/h)	24.1 ± 2.6	23.8 ± 2.4	0.4 ± 2.3	0.56
Mean HR (% of individual’s maximum)	76.7 ± 5.7	74.6 ± 6.7	1 ± 6.7	0.57
Max HR (% of individual’s maximum)	95.9 ± 5.1	94.6 ± 4.8	1.3 ± 3.7	0.22

*p* values were calculated using paired *t* test

**Fig. 2:**
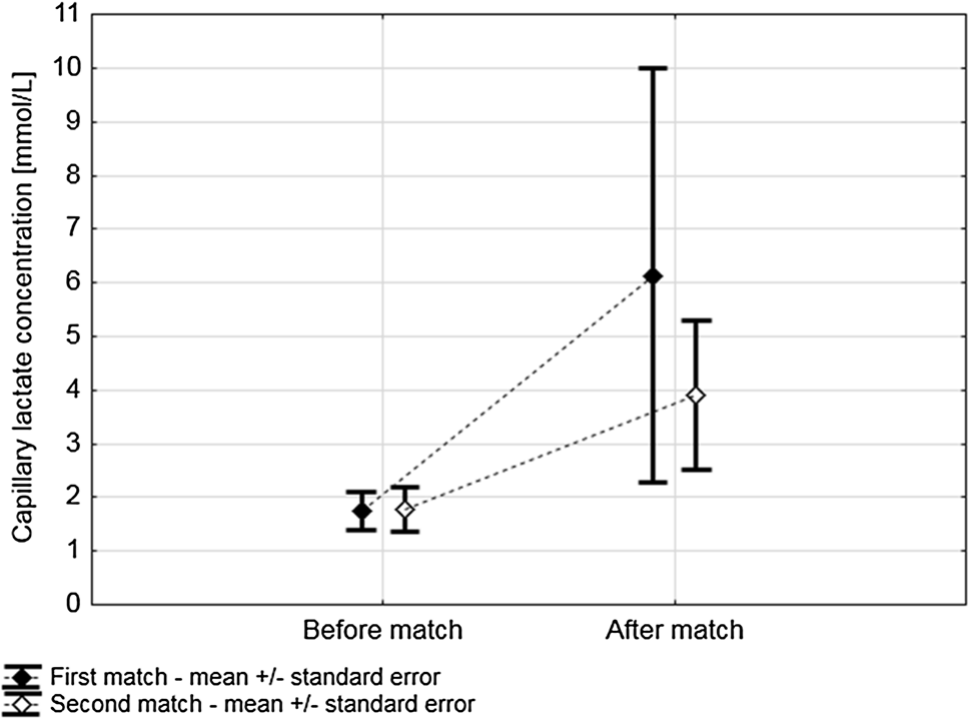
Changes in lactate concentrations during the first and second matches. During both matches, players presented similar mean lactate concentrations [Match 1: 4 mmol/l (SE = 0.78) vs. Match 2: 2.8 mmol/l (SE = 0.35), *p* = 0.23] with a significant rise during play [Match 1: 1.75 (SE = 0.16)–6.13 mmol/l (SE = 1.73); Match 2: 1.77 (SE = 0.18)–3.91 mmol/l (SE = 0.63), *p* = 0.004]. The rise of lactate was comparable between the events (*p* = 0.24). *SE* standard error of the mean

During both matches, a significant rise in capillary blood lactate was observed [mean baseline 1.76 mmol/l (SE: 0.15) to mean after matches 5.02 (SE: 0.96), *p* = 0.004]. For each player, the increase was similar in both matches (*p* = 0.24) (Fig. 2).

**Fig. 3:**
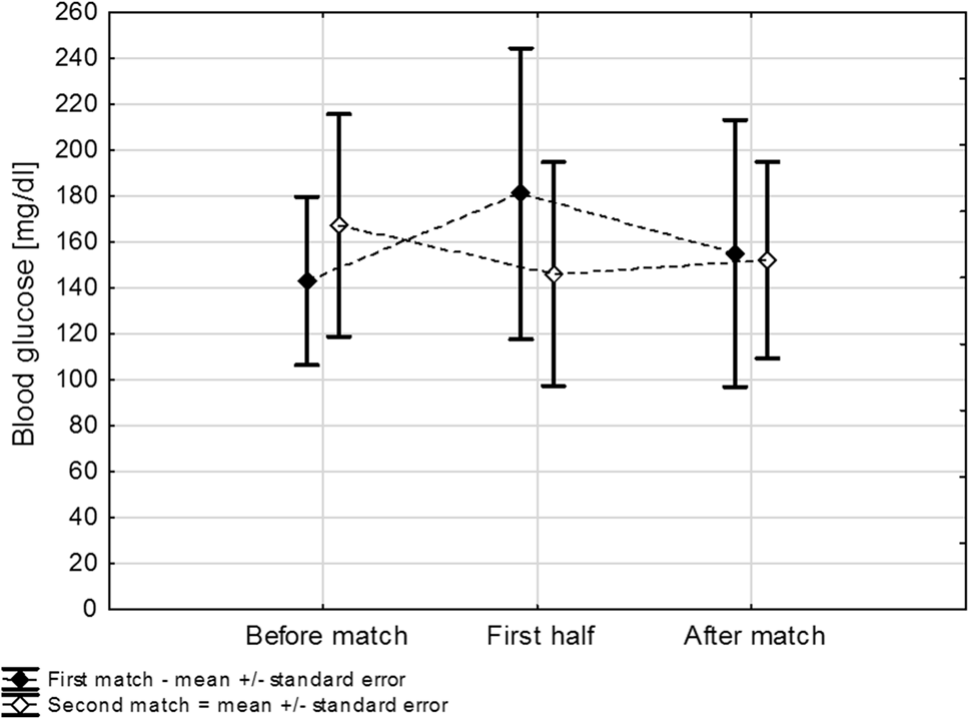
Changes in glycemia during the first and second matches. Mean blood glucose concentrations during both matches were comparable [Match 1: mean 160 mg/dl (SE: 14) vs. Match 2: 155 mg/dl (SE: 12), *p* = 0.83]. Overall, glycemia during both matches remained stable (*p* = 0.78); the differences between blood glucose dynamics between the matches were also not significant (*p* = 0.28). SE standard error of the mean

**Fig. 4:**
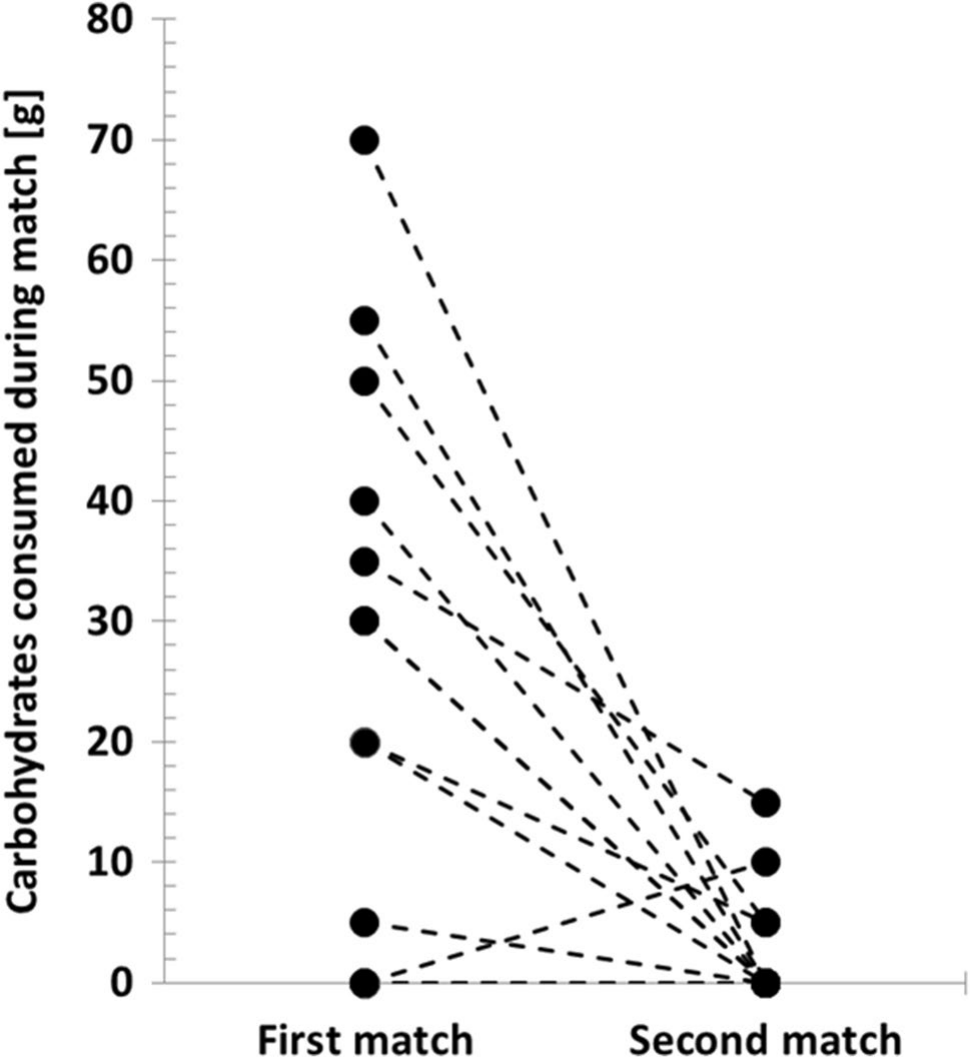
Carbohydrates consumed during the first and second matches. During the second match, individual players consumed significantly less carbohydrates [median difference − 20 g (25–75%: − 40 to 0), *p* = 0.006]

No significant differences in BG were observed when BG values before, at half of the match and after the match were compared between the two matches (*p* = 0.83) or when BG values before, at half and after were compared for each match individually (*p* = 0.78) (Fig. 3). Glucose alert values (≤ 70 mg/dl) were observed in four children during the first match and in two others during the second one (*p* for proportion = 0.01). Clinically significant hypoglycemia events (< 54 mg/dl) were noted in two players during the first match and in none during the second match. No player suffered severe hypoglycemia during the matches or over 24-h follow-up. CGM data were incomplete, which made statistical analysis impossible. Despite similar comparable workload characteristics of the matches, players consumed significantly less carbohydrates during the second match (median difference for each player: − 20 g [25–75%: − 40 to 0], *p* = 0.006) (Fig. 4).

No correlations were found between exercise- and diabetes-related parameters (Table 2).

**Table 2:** Correlations between exercise- and diabetes-related parameters

	First match						Second match					
	Mean HR	Max HR	Covered distance	Max velocity	Mean velocity	No. of sprints	Mean HR	Max HR	Covered distance	Mean velocity	Max velocity	No. of sprints
Age	*r* = 0.307	*r* = 0.266	*r* = 0.003	*r* = 0.624	*r* = 0.034	*r* = 0.351	*r* = 0.122	*r* = 0.157	*r* = 0.012	*r* = 0.060	*r* = 0.401	*r* = 0.232
	*p* = 0.286	*p* = 0.357	*p* = 0.992	*p* = 0.017	*p* = 0.908	*p* = 0.219	*p* = 0.677	*p* = 0.593	*p* = 0.967	*p* = 0.838	*p* = 0.155	*p* = 0.424
HbA1c	*r* = − 0.145	*r* = 0.107	*r* = − 0.339	*r* = − 0.037	*r* = − 0.321	*r* = − 0.260	*r* = 0.097	*r* = 0.276	*r* = − 0.198	*r* = − 0.159	*r* = 0.012	*r* = − 0.095
	*p* = 0.621	*p* = 0.715	*p* = 0.235	*p* = 0.899	*p* = 0.263	*p* = 0.370	*p* = 0.742	*p* = 0.340	*p* = 0.499	*p* = 0.588	*p* = 0.969	*p* = 0.746
Cooper *z*-score	*r* = 0.098	*r* = 0.121	*r* = 0.454	*r* = 0.080	*r* = 0.440	*r* = 0.235	*r* = 0.283	*r* = 0.101	*r* = 0.472	*r* = 0.453	*r* = − 0.082	*r* = 0.169
	*p* = 0.739	*p* = 0.681	*p* = 0.103	*p* = 0.787	*p* = 0.115	*p* = 0.418	*p* = 0.327	*p* = 0.708	*p* = 0.088	*p* = 0.104	*p* = 0.781	*p* = 0.563
BMI *z*-score	*r* = 0.348	*r* = 0.175	*r* = − 0.054	*r* = 0.308	*r* = − 0.013	*r* = 0.138	*r* = 0.070	*r* = 0.1474	*r* = − 0.187	*r* = − 0.138	*r* = 0.160	*r* = 0.214
	*p* = 0.223	*p* = 0.550	*p* = 0.855	*p* = 0.284	*p* = 0.965	*p* = 0.637	*p* = 0.812	*p* = 0.615	*p* = 0.523	*p* = 0.638	*p* = 0.585	*p* = 0.463

*r* Pearson’s correlation coefficient, *HR* heart rate

## Discussion

This is the first study to evaluate physical activity workload during football matches in T1DM adolescents with GPS tracking. Football is a mixed type, i.e., aerobic–anaerobic physical activity and the energy expenditure of an individual player may vary depending on age, physical fitness and position on the field [15]. Without a precise measurement of the obtained velocities, accelerations, and distances covered, assessment of players’ physical effort remains subjective and inaccurate [16, 17].

GPS tracking is used by professional sport clubs to estimate different aspects of workload during training sessions and matches. It also allows physiologists and sports medicine specialists to assess the fitness level of football players in order to better adapt personal training plans [18].

No studies assessed precisely the intensity of physical exertion during football matches in adult or adolescent T1DM patients so far. Thus, the only point of reference is the report on competitive adolescent players without diabetes [15]. Our patients covered about 75% of distance reported for competitive adolescent players without T1DM [15]. GPS tracking enabled us to accurately measure not only the covered distances, but also achieved velocities, accelerations and HR. Average HR obtained by the players indicated intense physical effort during both matches and corresponded well with the results obtained in healthy teenagers [19].

We showed that for each player both matches provided comparable workloads. Both matches could be considered as mixed aerobic–anaerobic activity periods based on the rise in capillary blood lactate concentrations [20]. Furthermore, a corresponding number of performed sprints suggest that anaerobic component was similar in both games. The partly anaerobic character of activity may explain why only isolated episodes of hypoglycemia were recorded during both matches, as it was shown that anaerobic exercise increases and rather not decreases BG levels. We must be also aware that the same physical effort can cause different glycemic changes in individual patients [10].

From the perspective of diabetes management, the sparring events differed only in the amount of simple carbohydrates consumed by players. This difference did not depend on any measured performance metrics. Thus, it can be speculated that smaller carbohydrate consumption during the second game could have resulted from the players’ adaptation to physical activity during the camp. In addition, the medical staff had more experience and less fear of hypoglycemia during the second match and could serve players somehow smaller simple carbohydrate portions (i.e., falling into the lower doses recommended by protocol). In both matches, the average amount of carbohydrates consumed was in the range recommended for mixed training lasting longer than 60 min under low blood insulin concentration conditions [21].

Overall, while the two matches provided comparable physical workloads, they also resulted in similar glycemic outcomes in individual players. Moreover, most of the BG values were in the target range set in the study protocol (100–250 mg/dl). This observation suggests that in case of young T1DM football players developing individual recommendations aimed at optimization of glycemic control during matches is possible based on the parameters of training units. Information on the intensity of the planned exercise or sport is important because individualized approach is required for modifications of insulin therapy proposed by diabetologists for periods of physical activity.

GPS tracking systems are not available for most football players on a daily basis. However, thanks to demonstrated repeatability of the matches, it may be possible to analyze a series of trainings or games and provide a player with customized guidelines. Furthermore, HR monitoring may be incorporated into algorithms that will modify the insulin delivery [22].

The strength of this study is that for the first time in patients with T1DM the GPS tracking was used during football matches, which enabled precise measurement of players’ physical activity workload. Performing both football matches in a short time interval and under the same general conditions provided an opportunity to better parameterize physical activity of individual players.

This study has some limitations. Firstly, not all football match-related activities can be tracked by GPS readings. Thus, workload associated with jumping, kicking a ball or tackling actions could only be reflected by HR monitoring [23]. We were also unable to perform glucose variability analysis based on CGM during the matches. The players used different types of continuous glucose monitoring systems, and moreover, the data from them were partially lost when the pumps were disconnected to prevent patients’ injury or equipment damage during games. Due to a small number of participants and differences in their characteristics (age, BMI, physical fitness, possibly also personal motivation for playing), the study lacked statistical power to pinpoint which measures of workload are most closely related to glycemic changes. A study including only competitive adolescent football players with T1DM is currently underway to mitigate this limitation.

Despite the difficulties in adjusting insulin therapy to physical exertion, children with diabetes should be encouraged to practice sports. The mixed nature of physical exercise, repetitive physical effort and low risk of hypoglycemia make football a sport that can be recommended for children and adolescents with T1DM. Furthermore, Cvetkovic et al. [24] showed that recreational football and high-intensity interval training elicited improvements in all muscular and cardiorespiratory fitness measures in adolescents, which may bring T1DM patients additional health benefits.

### Perspectives

Estimation of physical effort is essential for T1DM patients to properly adjust insulin dosage and carbohydrate intake to patient’s needs before, during and after practicing sports. One of the biggest challenges for young T1DM athletes is adaptation of therapeutic decisions to competitions, as the competition days may differ significantly from routine trainings due to the emotions and stress hormones interplay with glucose homeostasis. However, to our knowledge no study attempted to characterize glycemia dynamics during competitive or even semi-competitive play. Therefore, our most important result seems to be the observation that both matches were, for each individual, comparable in terms of kinematic parameters (distances, velocities), physical workloads (HRs), metabolic (lactate concentrations) and, to some extent, glycemic responses. This suggests that developing individual recommendations for each football player for the time of certain type of physical activity is feasible. Further, studies should be aimed at developing such personalized insulin therapy adaptation plans and testing them.

## Conclusions

HR monitoring coupled with GPS-based tracking effectively parameterized physical activity during a football match in T1DM children. T1DM patient workload and blood glucose changes during matches were comparable, which provides the opportunity to develop individual recommendations for athletes with T1DM. Adequate titration of carbohydrates before and during a football match can allow to keep BG within or close to the target range.

## Electronic supplementary material

Below is the link to the electronic supplementary material.

Supplementary Fig. 1 The figure contrasts workload parameters measured with GPS-tracking and HR-monitoring systems: A—distance covered; B—number of sprints; C—mean velocity; D—maximal velocity; E—mean HR; F—maximal HR. The comparisons were assessed using the paired t test and demonstrated no significant differences between the matches for individual players. For comparison of means and mean differences, refer to Table 1 in the main text. (PDF 211 kb)

Supplementary Fig. 2 Individual blood glucose measurements during first (A) and second (B) game. White bars indicate measurements before the match, green ones during the break and black ones immediately after the game. (TIFF 47 kb)

## Data Availability

The raw data supporting the conclusions of this manuscript will be made available by the authors, without undue reservation, to any qualified researcher.
