# Supplementary material for: Physical workload and glycemia changes during football matches in adolescents with type 1 diabetes can be comparable
Source: Acta Diabetol. 2019 Jun 4;56(11):1191–8. doi: 10.1007/s00592-019-01371-0 (PMC6768890; doi:10.1007/s00592-019-01371-0)
Supplement: Supplementary file 1 — The figure contrasts workload parameters measured with GPS-tracking and HR-monitoring systems: A—distance covered; B—number of sprints; C—mean velocity; D—maximal velocity; E—mean HR; F—maximal HR. The comparisons were assessed using the paired t test and demonstrated no significant differences between the matches for individual players. For comparison of means and mean differences, refer to Table 1 in the main text. (PDF 211 kb) [file 592_2019_1371_MOESM1_ESM.pdf]

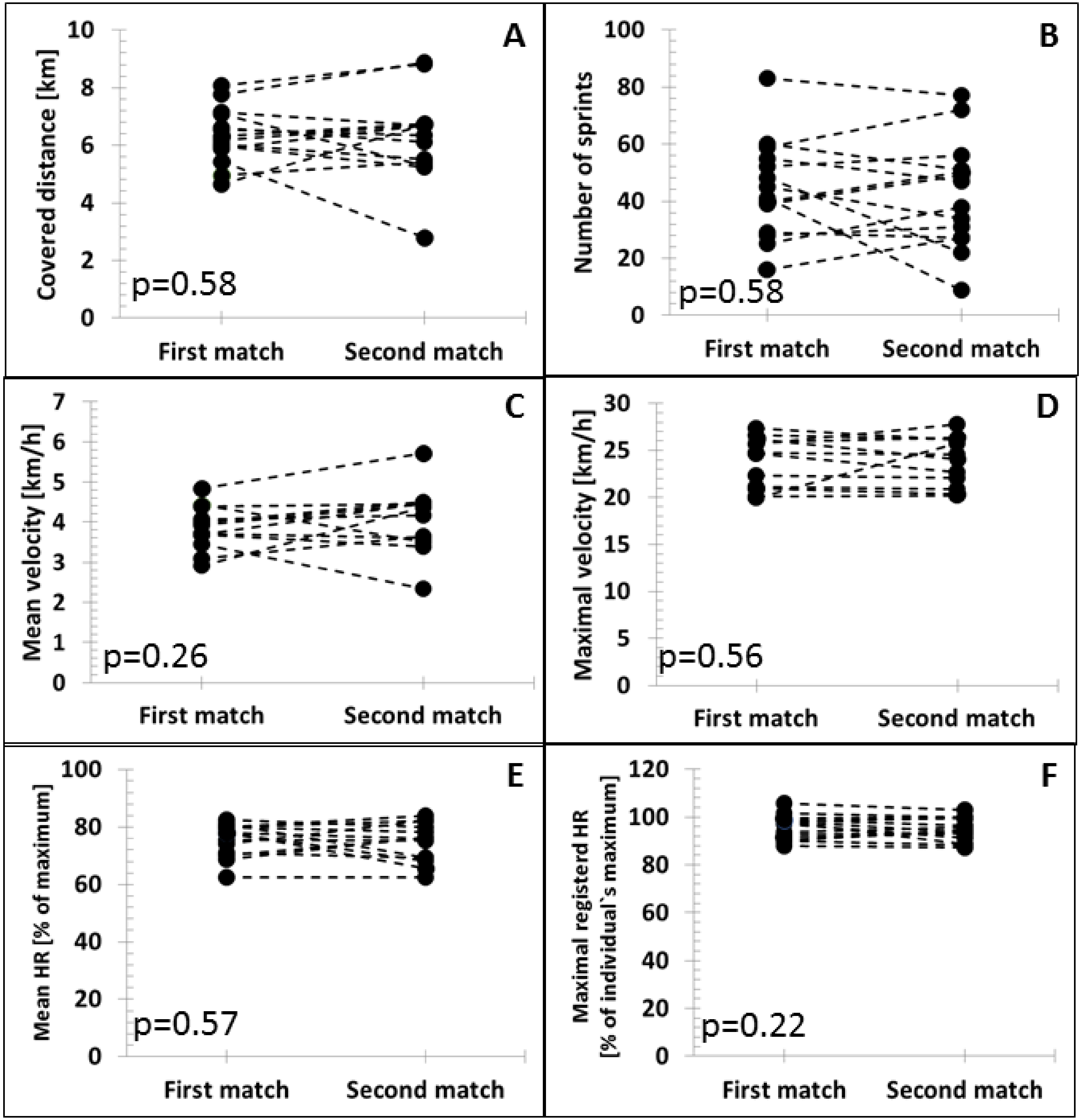
A
Covered distance [km]
p=0.58
First match
Second match
B
Number of sprints
p=0.58
First match
Second match
C
Mean velocity [km/h]
p=0.26
First match
Second match
D
Maximal velocity [km/h]
p=0.56
First match
Second match
E
Mean HR [% of maximum]
p=0.57
First match
Second match
F
Maximal registerd HR [% of individual`s maximum]
p=0.22
First match
Second match

Supplementary Figure 1. The figure contrasts workload parameters measured with GPS-tracking and HR-monitoring systems: A –distance covered; B – number of sprints; C – mean velocity; D – maximal velocity; E – mean HR; F – maximal HR. The comparisons were assessed using the paired t-test and demonstrated no significant differences between the matches for individual players. For comparison of means and mean differences, refer to Table 1 in the main text.
