# Supplementary figures and images for: Physical workload and glycemia changes during football matches in adolescents with type 1 diabetes can be comparable
Source: Acta Diabetol. 2019 Jun 4;56(11):1191–8. doi: 10.1007/s00592-019-01371-0 (PMC6768890; doi:10.1007/s00592-019-01371-0)

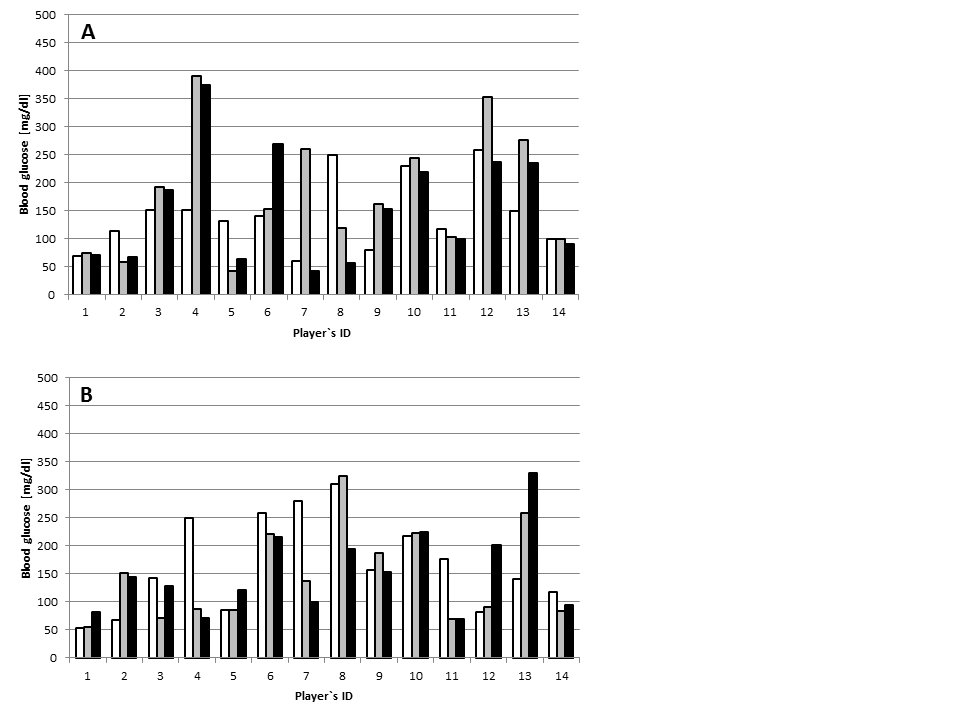

Supplement: Supplementary file 2 — Individual blood glucose measurements during first (A) and second (B) game. White bars indicate measurements before the match, green ones during the break and black ones immediately after the game. (TIFF 47 kb) [file 592_2019_1371_MOESM2_ESM.tif]
